# High-performance fractional order terminal sliding mode control strategy for DC-DC Buck converter

**DOI:** 10.1371/journal.pone.0187152

**Published:** 2017-10-30

**Authors:** Jianlin Wang, Dan Xu, Huan Zhou, Anning Bai, Wei Lu

**Affiliations:** 1 Department of Mechanical Electrical Engineering, Xi’an Jiaotong University, Xi’an, ShanXi, China; 2 Department of College of Science, Ningxia Medical University, Yinchuan, NingXia, China; Lanzhou University of Technology, CHINA

## Abstract

This paper presents an adaption of the fractional order terminal sliding mode control (AFTSMC) strategy for DC-DC Buck converter. The following strategy aims to design a novel nonlinear sliding surface function, with a double closed-loop structure of voltage and current. This strategy is a fusion of two characteristics: terminal sliding mode control (TSMC) and fractional order calculation (FOC). In addition, the influence of “the controller parameters” on the “performance of double closed-loop system” is investigated. It is observed that the value of terminal power has to be chosen to make a compromise between start-up and transient response of the converter. Therefore the AFTSMC strategy chooses the value of the terminal power adaptively, and this strategy can lead to the appropriate number of fractional order as well. Furthermore, through the fractional order analysis, the system can reach the sliding mode surface in a finite time. And the theoretical considerations are verified by numerical simulation. The performance of the AFTSMC and TSMC strategies is tested by computer simulations. And the comparison simulation results show that the AFTSMC exhibits a considerable improvement in terms of a faster output voltage response during load changes. Moreover, AFTSMC obtains a faster dynamical response, smaller steady-state error rate and lower overshoot.

## Introduction

DC–DC power converters are widely applied for supplying various output voltage in many electric vehicular systems, such as DC motor drives, the hybrid energy storage system (HESS), battery equalization and so on [1]. Our research team is mainly engaged in the research of HESS. The HESS contains two or more power sources connected by DC-DC converters. In order to improve the efficiency and performance of the HESS, the high-performance control strategy for DC-DC converters is needed. But DC-DC converters are inherently non-linear system with chaotic circuit. So the stability of the DC-DC converters is very important [2]. Therefore, the design of high-performance control strategy is usually a challenging issue.

The DC-DC converters in HESS are almost always multiple topologies including several MOSFET switches, so they are workable in both Buck mode and Boost mode. The power consumption of DC-DC converter is an important consideration for the HESS system, when compared with the power consumption of controller [3]. The power consumption of electronic components is a major part of the total power consumption in HESS. Therefore, we created the DC-DC converter structure with simplified design to reduce the power consumption. The control performance of DC-DC converter is studied in this paper. The control strategies of Buck and Boost converters have some similarities, so in this paper we just choose the Buck converter to investigate our novel control strategies.

The sliding mode control (SMC) has many advantages, such as its fast dynamic response, robustness to disturbances, guaranteed stability and simplicity in implementation [4]. There have been a lot of researches on sliding mode control for DC-DC converters. In Ref. [5], Hasan Komurcugil proposed an adaptive terminal sliding mode control strategy for Buck converter, and his sliding surface is a linear one based on linear combination of the system states, using an appropriate time-invariant coefficient. In Ref. [6], Yanmin Wang and her partner designed a double closed-loop structure for DC-DC converter feedback control, and the double closed-loop have a smaller steady-state error than others. In Ref. [7], Junxiao Wang and his partner investigated the performance of the nonlinear disturbance observers with the sliding mode control for Buck converter. In Ref. [8–11], the authors pointed out a fractional order calculation applied in DC-DC converter and control respectively.

In this paper, we focus on the high-performance control strategy for the DC-DC Buck converter, and propose a novel method of fractional order on terminal sliding mode control (FTSMC). Then utilize the method to design a novel nonlinear sliding surface function (based on the double closed-loop structure) that is a fusion of characteristics of TSMC and FOC [12–13].

The rest of the paper is organized as follows: Section 2 introduces the basic principles of the DC-DC Buck converter. Section 3 deals with the design of terminal sliding surfaces for DC-DC Buck converter. Section 4 conducts the design of nonlinear controllers for the Buck converter based on the fractional order calculation and the terminal sliding mode control. Section 5 shows simulation results and the adaptive methods to determine the terminal power parameter values. Section 6 states some conclusions and guidelines for further works.

## Modeling the DC-DC Buck converter

**Fig 1:**
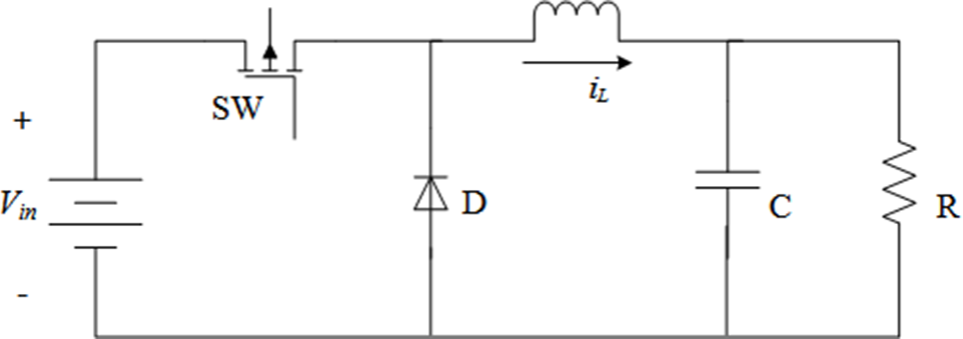
DC-DC Buck converter topology.

The topology of DC-DC Buck converter is shown in Fig 1, and it consists of a DC input voltage source, a MOSFET switch, a diode, an inductor, a capacitor and a load resistor. The average state equations describing the operation of the Buck converter can be written as

$$\frac{di_{L}}{dt}=\frac{1}{L}(uV_{in}-V_{o})$$ (1)

$$\frac{dV_{o}}{dt}=\frac{1}{C}(i_{L}-\frac{V_{o}}{R})$$ (2)

where *u* is the control input that takes 1 for the ON state of the switch, and 0 for the OFF state [14].

Let us define the output voltage error, *x*_1_ is

$$x_{1}=V_{o}-V_{ref}$$ (3)

Where *V*_*ref*_ is the reference value of the output voltage. By taking the time derivative of (3), *x*_2_ which is the rate of change of voltage error can be expressed as

$$x_{2}={\dot{x}}_{1}={\dot{V}}_{o}-{\dot{V}}_{ref}\approx {\dot{V}}_{o}$$ (4)

The state-space model of the Buck converter can be transformed to

$$\left[\begin{array}{c} {\dot{x}}_{1}\\ {\dot{x}}_{2} \end{array}\right]={\left[\begin{array}{cc} 0 & 1\\ -\frac{1}{LC} & -\frac{1}{RC} \end{array}\right]}_{}^{}\left[\begin{array}{c} x_{1}\\ x_{2} \end{array}\right]+\left[\begin{array}{c} 0\\ \frac{uV_{in}-V_{ref}}{LC} \end{array}\right]$$ (5)

## Terminal sliding mode control for the Buck converter

Most of the TSMC strategies are commonly used for the linear sliding surface, which is based on a linear combination of the system states, using an appropriate and time-invariant coefficient [15]. Therefore, the value of coefficient has to be chosen and this is an embarrassment [16–17]. Considering the confusion of this embarrassment, we designed a terminal sliding surface function with a double closed-loop structure of voltage and current. The function *S* can be defined as

$$S=i_{L}(t)-k_{a}x_{1}{}^{\gamma }-k_{b}\int_{0}^{t}x_{1}{}^{\gamma }d\tau$$ (6)

Where *k*_*a*_ > 0, *k*_*b*_ > 0, 0 < γ < 1, and they are positive odd integers. When the system is in the terminal sliding mode, it means the Eq (6) is equal to 0 (*S* = 0), assuming

$$y=\int_{0}^{t}x_{1}{}^{\gamma }d\tau$$ (7)

The system dynamics can be determined by the following nonlinear differential equation

$$k_{a}\dot{y}=i_{L}(t)-k_{b}y$$ (8)

Note that Eq (8) can also be written as

$$dt=\frac{k_{a}}{i_{L}-k_{b}y}dy$$ (9)

Taking integral of both side of Eq (9) and evaluating the resulting equation on the closed interval (*x*_1_(0) ≠ 0, *x*_1_(*t*_*s*_) = 0), the finite time *t*_*s*_ is obtained by

$$t_{s}=\left|-\frac{k_{a}}{k_{b}}\ln(i_{L}-\frac{k_{b}}{1+\gamma }x_{1}{\left(0\right)}^{1+\gamma })\right|$$ (10)

From Eq (10), it is obvious that the convergence time *t*_*s*_ still depends on the parameters *k*_*a*_, *k*_*b*_ and γ. Therefore, these parameters must be carefully selected to ensure the desired response.

The sufficient condition for the existence of the terminal sliding mode is given by

$$S\dot{S}<0$$ (11)

Select the Lyapunov function as

$$V=\frac{1}{2}S^{2}$$ (12)

The time derivative of Eq (12) can be written as

$$\dot{V}=S\dot{S}$$ (13)

In order to satisfy the Lyapunov function, the deviation from the terminal sliding surface *S* and its time derivative, $\dot{S}$ should be opposite signs in the vicinity of a sliding surface.

When *u* = 1, *S* < 0, so need $\dot{S}>0$

$$\dot{S}=\frac{1}{L}(V_{in}-V_{o})-k_{a}\cdot \gamma \cdot x_{1}{}^{\gamma -1}\cdot \frac{1}{C}(i_{L}-\frac{V_{o}}{R})-k_{b}x_{1}{}^{\gamma }>0$$ (14)

When *u* = 0, *S* > 0, so need $\dot{S}<0$

$$\dot{S}=-\frac{1}{L}V_{o}-k_{a}\cdot \gamma \cdot x_{1}{}^{\gamma -1}\cdot \frac{1}{C}(i_{L}-\frac{V_{o}}{R})-k_{b}x_{1}{}^{\gamma }<0$$ (15)

Make the value of γ, *i*_*L*_ approximately equal to 1 and 0 respectively, the conditions that limit the existence region of the design parameters are obtained as

$$0<k_{a}<\frac{CR}{L}$$ (16)

$$0<k_{b}\leq \frac{k_{a}}{CR}$$ (17)

From Eq (6) and $\dot{S}=0$, the equivalent control law *u*_*eq*_ in this case becomes

$$u_{eq}=\frac{L}{V_{in}}[\frac{k_{a}\cdot \gamma \cdot x_{1}{}^{\gamma -1}}{C}(i_{L}-\frac{V_{o}}{R})+k_{b}\cdot x_{1}{}^{\gamma }+\frac{V_{o}}{L}]$$ (18)

From expression Eq (18) using the constraint |*u*_*eq*_| ≤ 1, and considering the aforementioned equilibria conditions, the conditions that limit the existence region of the design parameters are obtained as

$$0<k_{a}<\frac{CR}{L}\frac{V_{in}-V_{ref}}{V_{ref}}$$ (19)

$$0<k_{b}\leq \frac{V_{in}}{LV_{ref}}$$ (20)

To solve the inequality Eqs (16), (17), (19) and (20), we should determine the parameters value approximately.

## Fractional order terminal sliding mode control for the Buck converter

The terminal sliding surface function is expressed as a fractional order differential equation that is obtained in the form

$$S=i_{L}(t)-k_{a}x_{1}{}^{\gamma }-k_{b}D_{0}^{-\lambda }x_{1}{}^{\gamma }$$ (21)

Where γ ∈ [0,1], λ ∈ [0,1], *k*_*a*_ *k*_*b*_ are positive constant. For the Buck converter with FTSMC, the time derivative of Eq (21) can be written as

$$\dot{S}={\dot{i}}_{L}(t)-k_{a}\cdot \gamma \cdot x_{1}{}^{\gamma -1}\cdot {\dot{x}}_{1}-k_{b}D_{0}^{1\text{-}\lambda }x_{1}{}^{\gamma }$$ (22)

Following the procedure of the previous section, the obtained expression for equivalent control is:

$$u_{eq}=\frac{L}{V_{in}}[\frac{k_{a}\cdot \gamma \cdot x_{1}{}^{\gamma -1}}{C}(i_{L}-\frac{V_{o}}{R})+k_{b}D_{0}^{1\text{-}\lambda }x_{1}{}^{\gamma }+\frac{V_{o}}{L}]$$ (23)

To obtain the sliding mode dynamics, we insert (21) into (5), and find that the whole closed loop system is in fractional order. Obviously, it is more appropriate to analyze the stability and convergence via the fractional version of Lyapunov by direct method [18–21].

Selecting the Lyapunov function as

$$V=S^{2}$$ (24)

It follows from the Ref. [22], if 0 is the equilibrium point of system (21) and *x*(0) = *x*_0_, the fractional order derivative of Eq (24), can be written as

$$\begin{array}{l} D^{1-\lambda }V=D^{-\lambda }\dot{V}\leq -KD^{-\lambda }\left\|x_{1}\right\|\\ =-Kl^{-1}D^{-\lambda }\left\|S\right\|\leq -Kl^{-1}\left\|D^{-\lambda }S\right\|\\ =-Kl^{-1}\left\|x_{1}\right\| \end{array}$$ (25)

Where K is positive constant, *l* is Lipschitz constant and *l* > 0. So, we can find *V* > 0 and *D*^1−λ^*V* < 0. In other words, the controlled system satisfies the reaching condition.

When the system reaches the sliding surface, which is *S* = 0, it is in the “terminal sliding” mode. Its dynamics can be determined by the following equation:

$$k_{a}x_{1}{}^{\gamma }=i_{L}(t)-k_{b}D_{0}^{-\lambda }x_{1}{}^{\gamma }$$ (26)

We know, several reputed definitions for fractional derivatives are put forward, including Riemann-Liouville definition, Grunwald-Letnikov definition, Caputo definition, Weyl definition, and Marchaud definition [23]. Among them, Riemann-Liouville definition has been well studied. So, we use Riemann-Liouville definition for fractional order differential operation as

$$k_{b}D_{0}^{1+\lambda }(D_{0}^{-1-\lambda }{\dot{x}}_{1}{}^{\gamma })=D_{0}^{1+\lambda }\left(i_{L}(t)-k_{a}x_{1}{}^{\gamma }\right)$$ (27)

Taking fractional integral of both side of Eq (27), the finite time *t*_*s*_ is obtained by

$$t_{s}=\left|-\frac{k_{b}\Gamma (\gamma +\lambda )}{k_{a}\Gamma (\gamma +1)}\ln(i_{L}-\frac{k_{b}x_{1}{\left(0\right)}^{2+\gamma -\lambda }}{\left(2+\gamma -\lambda \right)})\right|$$ (28)

Therefore, it can be concluded that system trajectories can reach the equilibrium point in a finite time. When λ = 1, it is obvious that (28) is equivalent to (10). It means that the finite time taken to attain the equilibrium point of the FTSMC system, is the same as the one of the TSMC system, as given in (10).

## Adaptive strategy and simulation results

In order to show the performance of the FTSMC, the DC-DC Buck converter system was subsequently tested by simulations. Simulations are carried out using MATLAB/Simulink. The Simulation framework is shown in S1 Fig, and parameters of Buck converter are given in Table 1.

**Table 1:** Specifications of Buck converter.

Descriptions	Parameters	Nominal values
Input voltage	V_in_	25V
Desired output voltage	V_ref_	10V
Inductance	L	260 uH
Capacitance	C	100 uF
Load resistance	R	1~10Ω

**Fig 2:**
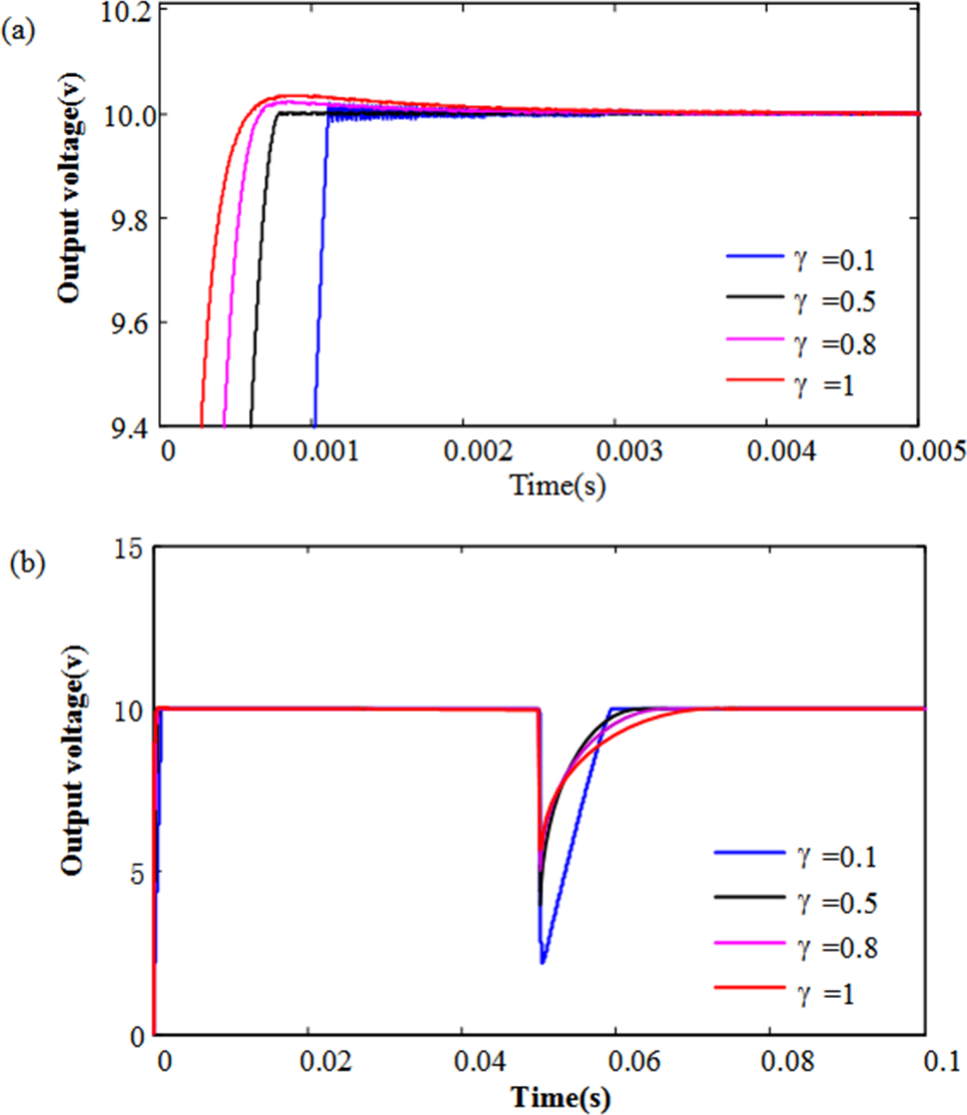
The output voltage dynamic response with different γ. (a) The output voltage dynamic response in start-up; (b) The output voltage dynamic response during load variations.

From Table 1 and the Eqs (16), (17), (19) and (20), we chose *k*_*a*_ = 0.8 *k*_*b*_ = 780, and the value range of terminal power (γ) is between 0 and 1, the performance of the proposed integer-order terminal sliding mode control strategy are showed in Fig 2.

It is clear from Fig 2(A) that the output voltage responses at the start-up become faster with increasing the value of γ. But the large value of γ can make overshoots and take a long time to reach the equilibrium point of the Eq (10). Fig 2(B) shows the responses of the output voltage for step changes in R (from 10 Ω to 1Ω), which are obtained by the SMC method with γ = 1, and the TSMC method with different γ values. Unlike the start-up case, it is interesting to note that the output voltage responses become faster with decreasing the value of γ. Therefore, the value of γ is chosen as some constant, to make a compromise between start up and transient responses of the converter.

When *x*_1_ is near the equilibrium point, it can be seen as |*x*_1_| < 1, the *γ* leads to |*x*_1_^γ^| > |*x*_1_|. In such a case, the system state with the nonlinear term *x*_1_^γ^ converges toward equilibrium point faster than the linear term *x*_1_. On the other hand, when |*x*_1_| > 1, the *γ* leads to |*x*_1_^γ^| < |*x*_1_|, it means the system state with the nonlinear term *x*_1_^γ^ converges toward equilibrium point slower than the linear term *x*_1_.

So, we proposed the adaptive law to choose the value of γ, which builds a monotone increasing function *x*_1_ for γ. This function will choose the value of γ approximately equal to 1 when |*x*_1_| > 1, and choose the value of γ much smaller but not less than 0.25 when |*x*_1_| < 1. According to the boundary conditions and the Simulink results, we use MATLAB/CFTOOL to fit the function of *x*_1_ for γ, describe it as

$$\gamma =\frac{1}{\pi }\arctan(x_{1}-0.99)+0.5$$ (29)

**Fig 3:**
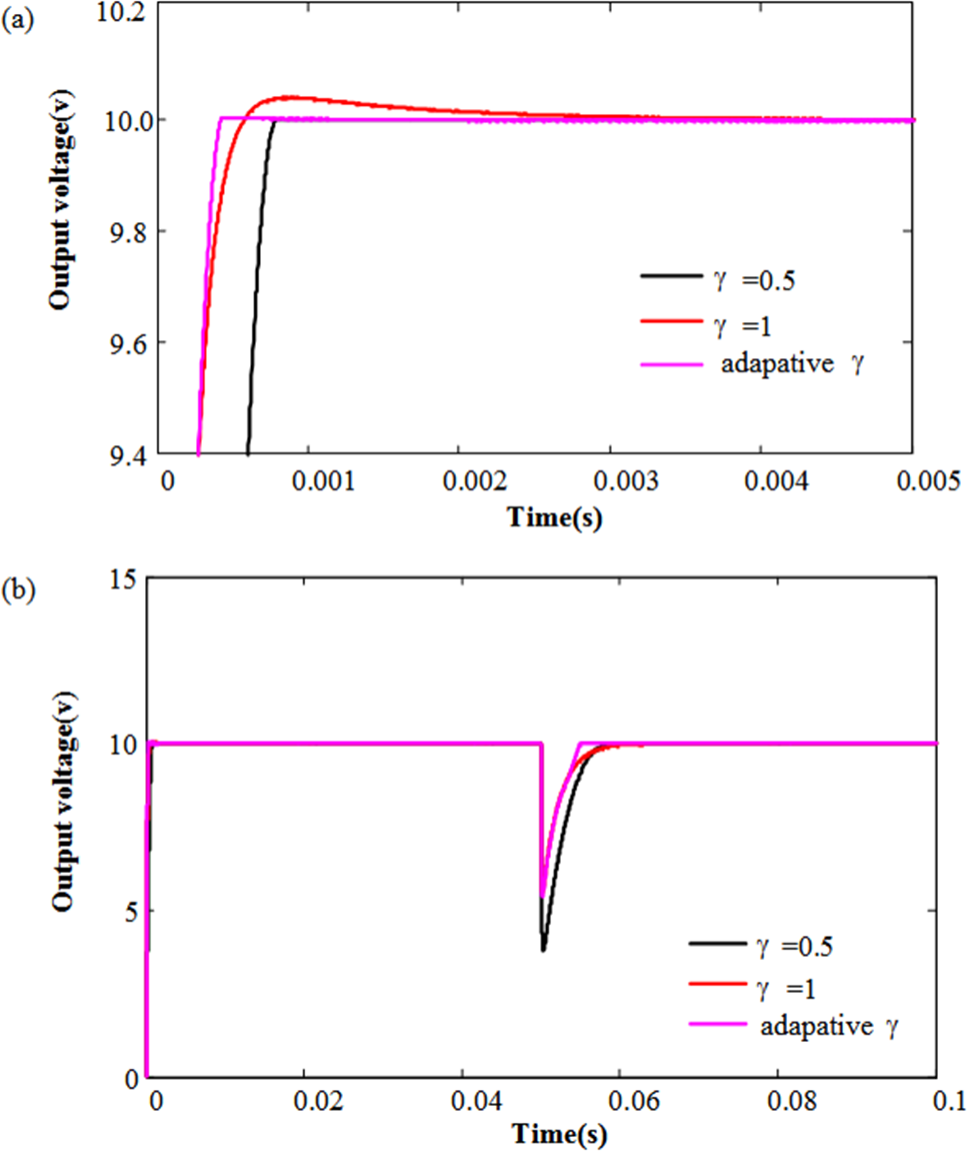
The output voltage dynamic response with adaptive γ. (a) The output voltage dynamic response in start-up; (b) The output voltage dynamic response during load variations.

From Eq (29), according to the state error, γ is selected adaptively, the simulation result as shown in Fig 3. We can easily observe the dynamic performance of the adaptive strategy better than the other constant terminal power.

**Fig 4:**
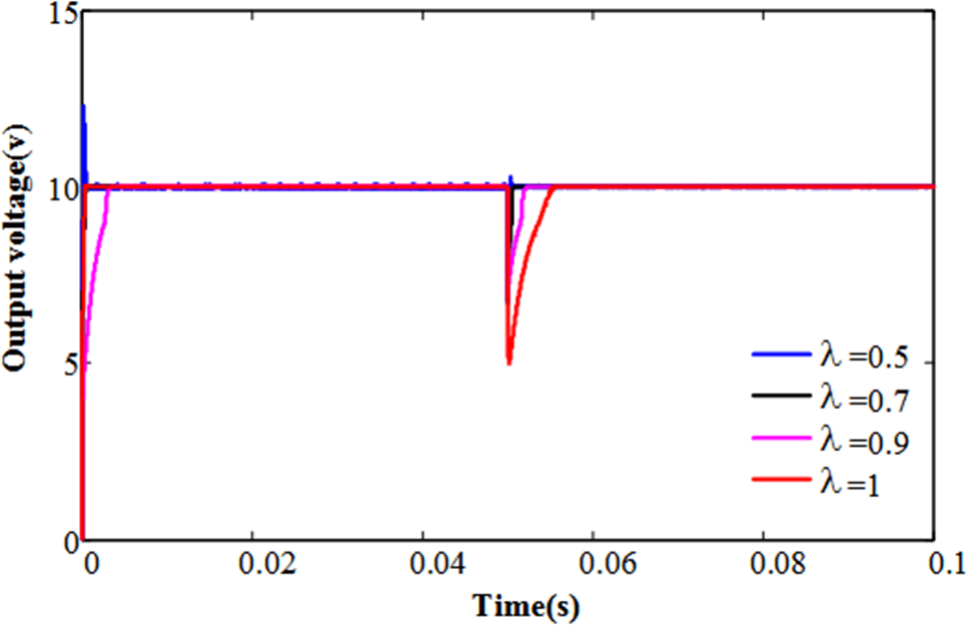
The output voltage dynamic response with different λ.

Further, we investigate the dynamic response of output voltage with different fractional order (*λ*) on the basis of the adaptive terminal sliding mode control strategy. Fig 4 shows the simulated start-up and transient responses of the output voltage obtained by AFTSMC strategies with different λ values. It is interesting to note that the output voltage responses become faster with decreasing the value of λ, but when λ = 0.5, the overshoot of the system appears and exceeds 25%. In order to obtain high performance control strategy, we should try to avoid the voltage overshoot and chattering. So choosing λ = 0.7 is our choice for ideal parameter value.

**Fig 5:**
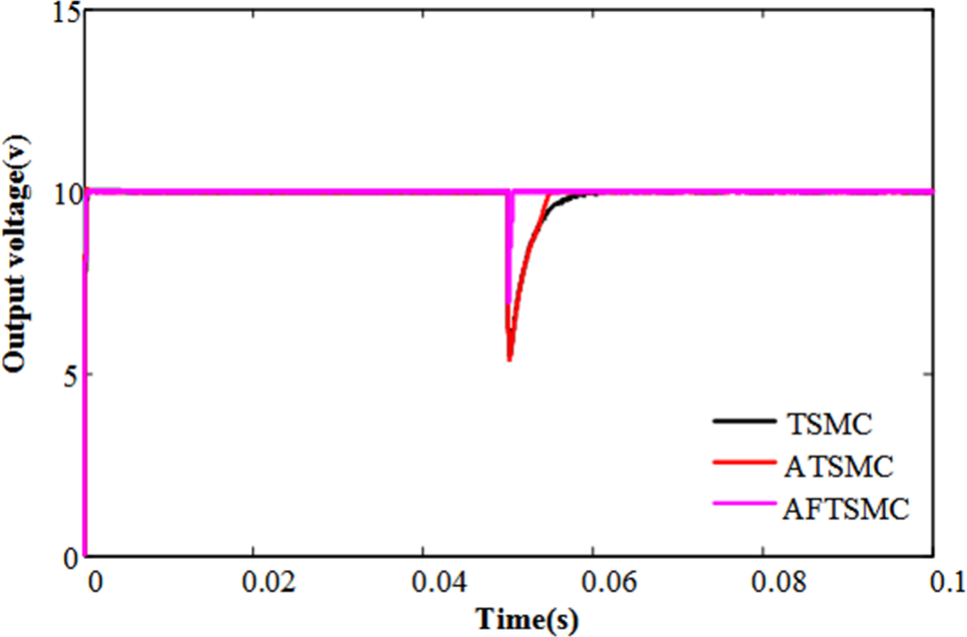
The output voltage dynamic response with different strategy.

In order to compare the control effect of TSMS, ATSMC, and AFTSMC, we use the parameter selection as shown in Table 2. As shown in Fig 5, the response time of the system with AFTSMC is less than others. At t = 0.05 s, the load resistance is changed from 10Ω to 1 Ω. Therefore, the output current will be increased, and the output voltage has a short step-down. It can be seen that the output voltage returns faster to reference output voltage in AFTSMC.

**Table 2:** Controller parameters of the proposed methods.

Descriptions	*k*_*a*_	*k*_*b*_	γ	λ
SMC	0.8	780	1	1
ATSMC	0.8	780	adaptive	1
AFTSMC	0.8	780	adaptive	0.7

## Conclusions

The fractional order terminal sliding mode control (FTSMC) based on a double closed-loop structure of voltage and current has been proposed. The influence of the controller parameters was investigated. It is observed that the chosen value of terminal power aims to make a compromise between the start-up and the moment when load changes. For this matter, we proposed an adaptive law to choose the terminal power, and the simulation shows that the method is effective. Further, we investigated the dynamic response of output voltage with different fractional orders, on the basis of the adaptive terminal sliding mode control strategy. It is shown that when the fractional order (*λ*) equal to 0.7, the performance of dynamic responses is better than others. In addition, the simulation results show that the AFTSMC strategy has the better performance in comparison with the ATSMC and TSMC. The novel fractional terminal sliding mode control exhibits considerable improvement in terms of a faster output voltage response, in the start-up and during load changes.

## Supporting information

S1 Fig Simulation framework diagram. In this simulation framework, the sliding surface function *S* could be the integer or fractional order terminal sliding surface function.
(TIF)

S2 Fig The output voltage dynamic response with different strategy. The control strategies include terminal sliding mode control (TSMC), adaptive terminal sliding mode control (ATSMC), and adaptive fractional order terminal sliding mode control (AFTSMC) respectively.
(TIF)
