# Supplementary figures and images for: High-performance fractional order terminal sliding mode control strategy for DC-DC Buck converter
Source: PLoS One. 2017 Oct 30;12(10):e0187152. doi: 10.1371/journal.pone.0187152 (PMC5662226; doi:10.1371/journal.pone.0187152)

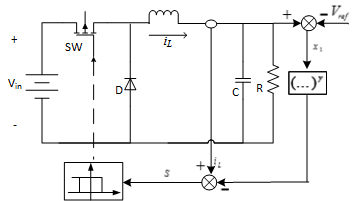

Supplement: S1 Fig — In this simulation framework, the sliding surface function S could be the integer or fractional order terminal sliding surface function. (TIF) [file pone.0187152.s001.tif]

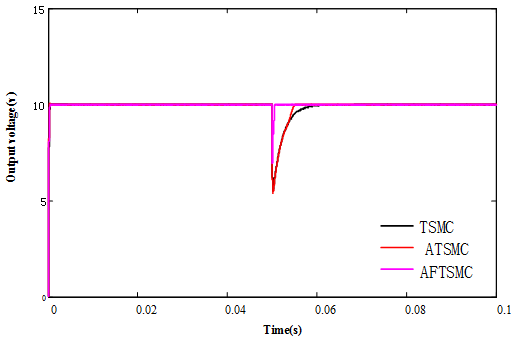

Supplement: S2 Fig — The control strategies include terminal sliding mode control (TSMC), adaptive terminal sliding mode control (ATSMC), and adaptive fractional order terminal sliding mode control (AFTSMC) respectively. (TIF) [file pone.0187152.s002.tif]
